# Matrix metalloproteinase 2 (MMP-2) and its tissue inhibitor 2 (TIMP-2) in pancreatic cancer (PC)

**DOI:** 10.18632/oncotarget.26571

**Published:** 2019-01-08

**Authors:** Marta Łukaszewicz-Zając, Mariusz Gryko, Sara Pączek, Maciej Szmitkowski, Bogusław Kędra, Barbara Mroczko

**Affiliations:** ^1^ Department of Biochemical Diagnostics, Medical University of Białystok, Białystok, Poland; ^2^ Second Department of General Surgery, Medical University of Bialystok, Bialystok, Poland; ^3^ Department of Neurodegeneration Diagnostics, Medical University of Białystok, Białystok, Poland

**Keywords:** biomarker, matrix metalloproteinases, pancreatic cancer

## Abstract

**Objectives:**

The incidence rate of pancreatic cancer (PC) is similar to mortality rate, thus searching specific tumor biomarkers of PC is sorely needed. Matrix metalloproteinase-2 (MMP-2) and the imbalance between MMP-2 and its tissue inhibitor (TIMP-2) play a critical role in tumor progression. We aim to assess the diagnostic and prognostic usefulness of serum MMP-2 and TIMP-2 as potential biomarkers in comparison to well-established tumor markers of PC (CA 19-9, carbohydrate antigen 19-9 and CEA, carcinoembryonic antigen).

**Results:**

We indicated the significant differences between serum TIMP-2 concentrations in PC patients, CP individuals and control group. The diagnostic sensitivity of TIMP-2 was the highest among all proteins tested and increased up to 96% in combined measurement with MMP-2. The area under ROC curve (AUC) for TIMP-2 was larger than for MMP-2, but lower than for classical tumor markers.

**Methods:**

Presented study comprised on 226 subjects, including 92 PC patients, 43 chronic pancreatitis (CP) patients and 91 healthy volunteers. The serum concentrations of these proteins were measured using immunological methods.

**Conclusions:**

Presented findings suggest higher usefulness of TIMP-2 than MMP-2 as potential biomarker in the diagnosis of PC patients, however more studies on large population are needed to support our results.

## INTRODUCTION

Pancreatic cancer (PC) is one of the most lethal solid tumor disease. This malignancy is ninth the most frequent tumor and the fourth most common cancer cause related deaths [1]. The patients with PC have extremely unfavorable prognosis, thus almost all of new cases of PC are expected to die from this disease. The poor prognosis is the result of the aggressive biological characteristics of tumor, such as local invasion and distant metastasis, which occur in early stage of disease [2].

Despite the improvement in the management of PC patients, the most effective treatment is early radical resection of tumor, however the five-year survival rate of PC patients is estimated as only 25–30% [3, 4]. Early diagnosis of malignant disease, including PC represents still serious problem for clinicians, especially in differentiation between pancreatic neoplasms and benign lesion. About 85% of patients with this malignancy have reached the advanced stage at first diagnosis, and in these cases the five-year survival rate is only 1–2% [4]. Average the five-year survival rate is the lowest of all gastrointestinal malignancies [5]. Despite the modern diagnostic methods, including imaging methods such as computed tomography, endoscopic ultrasound, endoscopic retrograde cholangiopancreatography, magnetic resonance imaging and magnetic resonance cholangiopancreatography have a limitation in the detecting of early-stage of PC [6]. In addition, the common use of fine needle biopsy in the differentiation between malignant and benign changes in pancreas may provide false negative results [6–8]. Therefore, novel methods of PC diagnosis and prognosis are critically needed in routine practice.

The great challenge of future medicine is to find a specific biochemical marker, that will be easy to use, cheap, non-invasive tool useful in early diagnosis of malignant disease, including PC. Carbohydrate antigen 19-9 (CA 19-9) has been validated as the first line serum marker, because of its high positive predictive value in PC patients. However, the diagnostic sensitivity and specificity of CA 19-9 measurement is not high enough to be use in early stage of PC, thus the diagnosis of PC patients in early stage of disease is crucial to improve clinical outcome [3, 7].

Matrix metalloproteinases (MMPs) are proteolytic enzymes responsible for the degradation of extracellular matrix (ECM) components as well as the basal membrane (BM) of the vessels. MMPs are involved in many physiological and pathological processes. Some clinical investigations have suggested that these enzymes might also play a potential role in the pathogenesis of chronic pancreatitis (CP) [9–12]. It has been proved that tumor progression, as a multi-step process, requires breakdown of ECM. Matrix metalloproteinase 2 (MMP-2) degrades type IV collagen, thus this gelatinase together with other gelatinase – matrix metalloproteinase 9 (MMP-9) plays the most important role in the invasion, migration and metastasis of neoplastic cells. Moreover, this enzyme promotes cleavage of ECM proteins and is intensively expressed in tumor and stromal components of many malignancies, including PC [9–12]. The degradation of ECM and BM via MMPs is regulated at several levels, including tissue inhibitors of metalloproteinases (TIMPs). Imbalance between these proteolytic enzymes and their tissue inhibitors leads to excessive BM and ECM degradation, facilitating the spread of cancer cells and neoangiogenesis [12]. The immunohistochemical study of Zhai *et al.* has revealed that the expression of MMP-2 was significantly higher in PC tissue in comparison to non-tumorous tissues [11]. In addition, the overexpression of MMP-2 in PC tissue positively correlated with higher preoperative serum CA19-9 levels, advanced stage, poor histological grade, lymph node matastasis, perineural invasion, and distant metastasis [11]. The enzymatic activity of MMP-2 was also determinated in all tissue samples of PC and CP, what was confirmed using zymography analysis [13]. In the literature, there are few reports on the blood concentrations of MMP-2 and TIMP-2 [9, 10]. However, according to our knowledge, little is known about diagnostic usefulness of MMP-2 in relation to its tissue inhibitor in the sera of PC patients and in comparison to well-established tumor markers, such as CA 19-9 and CEA. Thus, the aim of our study was to evaluate the clinical significance of these proteins as potential tumor biomarkers for PC, based on diagnostic characteristics, such as diagnostic sensitivity and specificity, accuracy, predictive values for negative (NPV) and positive (PPV) results as well as the areas under the ROC curve (AUC). Moreover, the correlations between serum levels of analyzed proteins and the clinico-pathological characteristics of tumor as well as resectability and survival of PC patients were also assessed. We expect that serum MMP-2 and its tissue inhibitor (TIMP-2) levels will be significantly different in PC patients in comparison to healthy volunteers and the measurement of these proteins concentrations might be useful in the diagnosis and progression of PC. Present study is the continuation of our previous investigations, where we assessed whether the serum levels of MMP-2 and TIMP-2 might be used as potential tumor markers for gastric (GC), esophageal (EC) and colorectal cancer (CC) [14–16]. Moreover, previously we also indicated the diagnostic and prognostic utility of other gelatinase (matrix metalloproteinase 9, MMP-9) and its tissue inhibitor 1 (TIMP-1) in PC [17].

## RESULTS

The serum levels of all proteins tested in PC and CP patients as well as in healthy individuals are presented in Table 1. The serum levels of TIMP-2 in PC patients were significantly lower compared to healthy controls, but statistically higher than in patients with CP (*p* < 0.001) (Table 1). The concentrations of classical tumor markers (CA 19-9 and CEA) were higher in PC patients when compared to CP individuals and healthy controls and all these differences were statistically significant (*p* < 0.001).

**Table 1:** Serum levels of proteins tested in patients with pancreatic cancer (PC) in comparison to chronic pancreatitis (CP) patients and healthy controls

		CEA (ng/mL)				CA19-9 (U/mL)				MMP-2 (ng/mL)				TIMP-2 (ng/mL)			
		Median		Range	*p*	Median		Range	*p*	Median		Range	*p*	Median		Range	*p*
**Group tested**	Pancreatic cancer	2.7^AB^	0.6	884.0	<0.001^*^	190.2^AB^	0.0	50000.0	<0.001^*^	223	122	496	0.080	88^AB^	45	140	<0.001^*^
	Chronic pancreatitis	1.4^A^	0.1	43.5		5.7^A^	0.0	1548.5		236	126	447		78^A^	30	148	
	Healthy controls	0.8	0.1	11.4		0.8	0.0	52.9		205	118	384		94	54	162	
**Tumor stage (TNM)**	II	1.6	0.7	7.4	0.013^*^	168.8	3.8	15003.0	0.869	183	149	381	0.611	89	67	105	0.923
	III	3.6	1.1	110.7		147.2	0.0	44792		240	131	407		88	45	137	
	IVA	1.9	0.7	884.0		165.6	0.0	5742.9		240	127	496		88	62	125	
	IVB	4.6 ^C^	0.6	320.5		310.1	0.0	50000.0		224	122	458		86	55	140	

A - statistically significant when compared to healthy subjects in Dwass-Steel-Critchlow-Fligner post hoc test.

B - statistically significant when compared to chronic pancreatitis patients in Dwass-Steel-Critchlow-Fligner post hoc test.

C - statistically significant when compared to stage II in Dwass-Steel-Critchlow-Fligner post hoc test.

The relationship between serum levels of analyzed proteins and clinico-pathological parameters of tumor was presented in Table 1. Serum concentrations of MMP-2 and TIMP-2 did not correlate with TNM stage. The statistically significant difference between classical tumor markers levels and TNM stage was found only for CEA concentrations (*p* = 0.013).

Serum levels of MMP-2 were elevated in PC patients with nodal involvement (N1 subgroup), presence of distant metastasis (M1 subgroup) and high depth of tumor invasion (T3 and T4 subgroups) in comparison to individuals from T2, N0 and M0 subgroups. The serum levels of TIMP-2 concentrations were lower in more advanced clinical stage (N1 and M1 subgroups) of PC, but there were also no significant associations between median concentrations of TIMP-2 and clinico-pathological characteristic of tumor. The concentrations of CEA concentrations were significantly higher in patients with larger depth of tumor invasion, presence of lymph node and distance metastases when compared to those of T2, N0 and M0 subgroups (Table 2).

**Table 2:** Serum levels of proteins tested in patients with pancreatic cancer (PC) in relation to clinico-pathological characteristics of tumor

		CEA (ng/mL)				CA19-9 (U/mL)				MMP-2 (ng/mL)				TIMP-2 (ng/mL)			
		Median		Range	*p*	Median		Range	*p*	Median	Range		*p*	Median		Range	*p*
**Tumor size (T factor)**	T2	1.4	0.7	5.3	0.005^*^	46.8	0.0	15003.0	0.260	183	161	381	0.330	88	67	137	0.372
	T3	3.7^A^	0.7	110.7		252.5	13.5	44792.0		262	131	458		92	45	140	
	T4	3.0^A^	0.6	884.0		218.5	0.0	50000.0		237	122	496		86	55	127	
**Nodal involvement (N factor)**	N0	1.7	0.7	14.6	0.015^*^	80.7	0.0	15003.0	0.324	195	127	381	0.325	89	60	112	0.829
	N1	3.5	0.6	884.0		240.1	0.0	50000.0		240	122	496		88	45	140	
**Distant metastases (M factor)**	M0	2.1	0.7	884.0	0.004^*^	165.6	0.0	44792.0	0.493	221	127	496	0.895	89	45	137	0.656
	M1	4.6	0.6	320.5		310.1	0.0	50000.0		224	122	458		86	55	140	
**Resectability**	Resectable	1.9	0.7	103.7	0.006^*^	74.7	0.0	15003.0	0.029^*^	207	129	458	0.257	89	45	137	0.933
	Nonresectable	3.7	0.6	884.0		310.1	0.0	50000.0		243	122	496		86	55	140	
**Survival of patients**	Alive	1.2	0.7	14.6	0.004^*^	65.4	0.0	635.8	0.103	240	158	381	0.498	91	62	111	0.504
	Dead	4.5	0.6	884.0		310.1	0.0	44792.0		261	160	496		88	55	125	

A - statistically significant when compared to T2 tumors in Dwass-Steel-Critchlow-Fligner post hoc test.

^*^statistically significant when *p* < 0.05.

If we assessed the relationship between serum concentrations of analyzed proteins and resectability of PC, only serum CEA and CA 19-9 levels were significantly lower in patients with resectable tumor in comparison to nonresectable PC. Moreover, we indicated that serum levels of MMP-2 and classical tumor markers were lower, whereas TIMP-2 concentrations – higher in PC who alive in comparison to those who died because of PC, however the significant difference was found only for CEA concentrations (*p* = 0.004, Table 2).

Associations between serum MMP-2, TIMP-2, CA 19-9 and CEA levels and prognosis of PC patients’ survival were assessed using univariate analysis. We demonstrated that tumor stage (*p* = 0.002), nodal involvement (*p* = 0.031) and the presence of distant metastases (*p* < 0.001) as well as resectability of tumor (*p* = 0.004) were significant factors affecting PC patients’ survival. None of proteins tested were significant prognostic indicators. Multivariate regression analysis with Cox’s proportional hazard model indicated that tumor stage (*p* = 0.018), the presence of distant metastases (*p* = 0.007) and resectability of tumor (*p* = 0.028) were proved to be independent prognostic factors of PC patients’ survival (data not shown).

**Figure 1:**
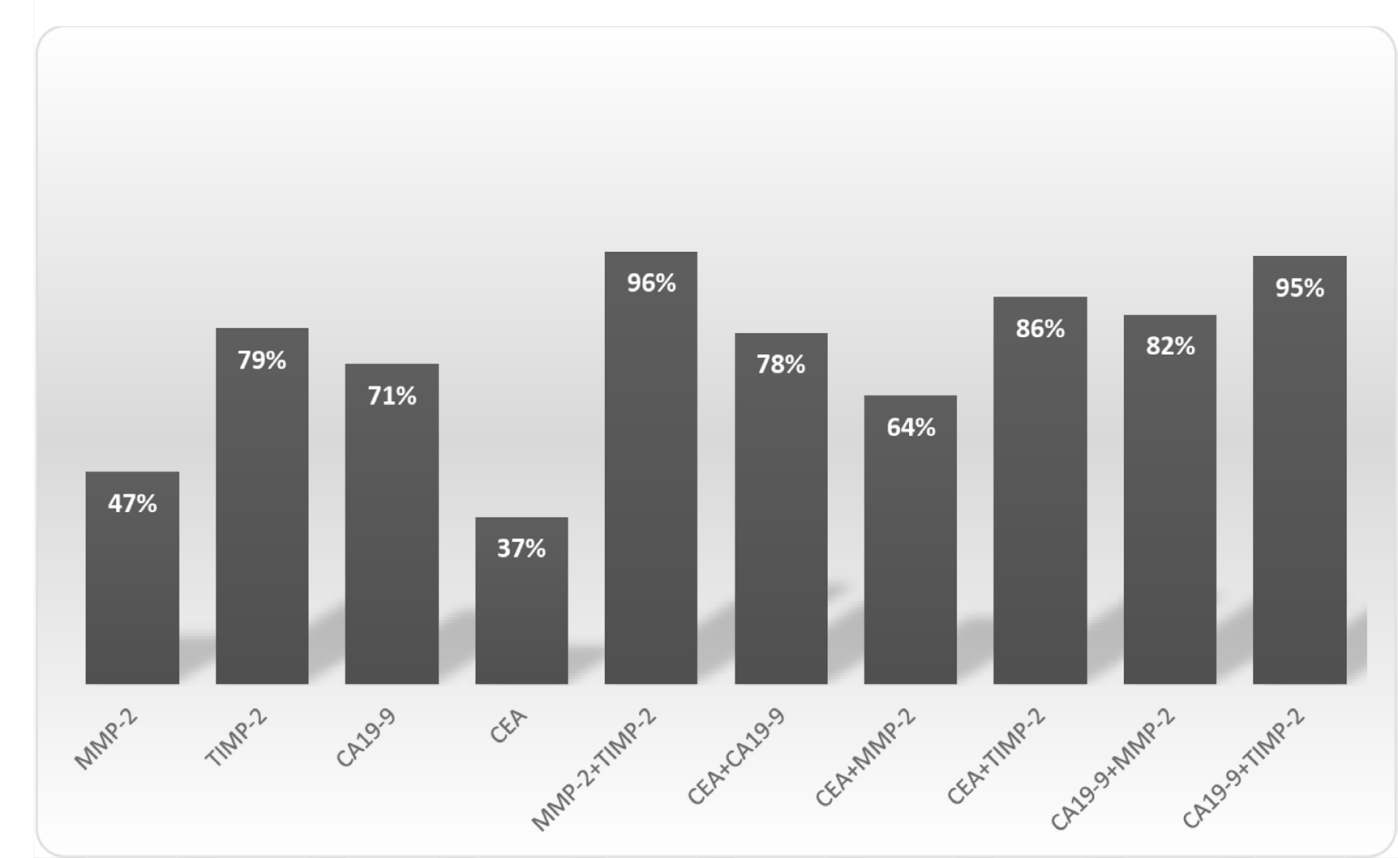
The diagnostic sensitivity of MMP-2 and its tissue inhibitor TIMP-2 as well as classical tumor markers in pancreatic cancer (PC) patients

**Figure 2:**
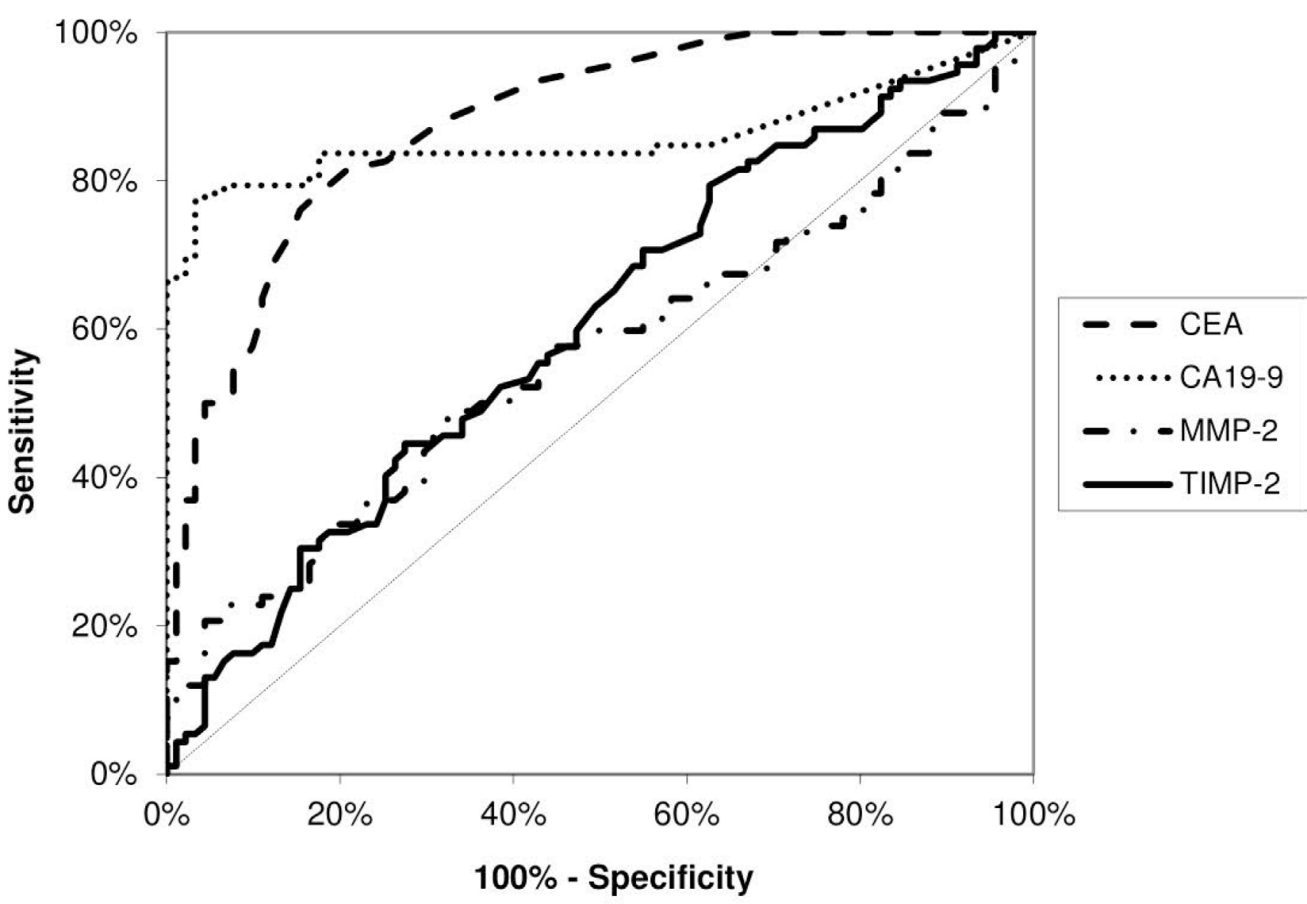
Areas under ROC curve (AUC) for TIMP-2 (0.6037, *p* = 0.013), MMP-2 (0.5624, *p* = 0.146), CA 19-9 (0.8583, *p* < 0.001) and CEA (0.8790. *p* < 0.001) in the differentiation between pancreatic cancer patients and healthy subjects

**Figure 3:**
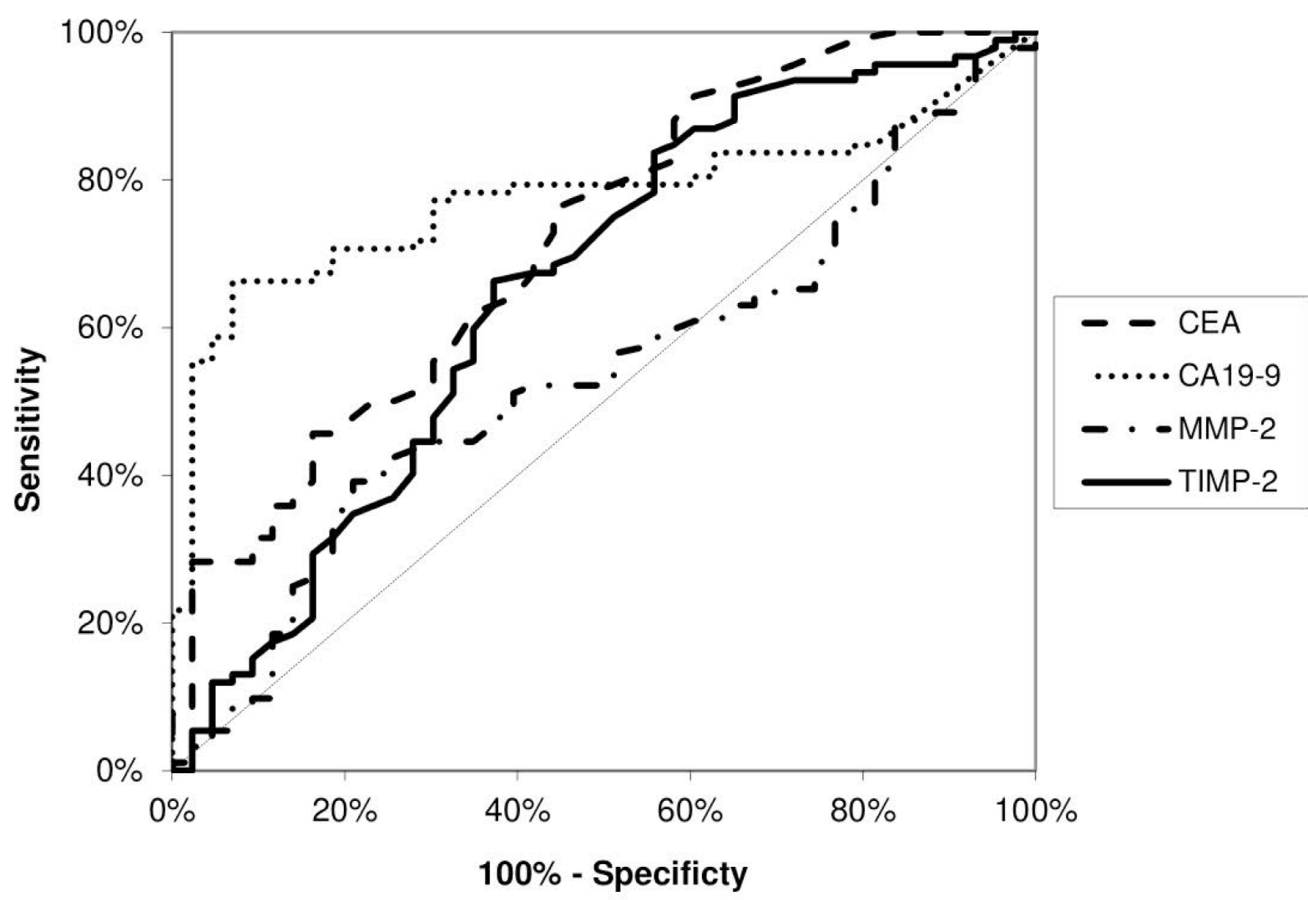
Areas under ROC curve (AUC) for TIMP-2 (0.6532, *p* = 0.004), MMP-2 (0.5378, *p* = 0.469), CA 19-9 (0.7765, *p* < 0.001) and CEA (0.7193, *p* < 0.001) in the differentiation between pancreatic cancer patients and chronic pancreatitis patients

**Figure 4:**
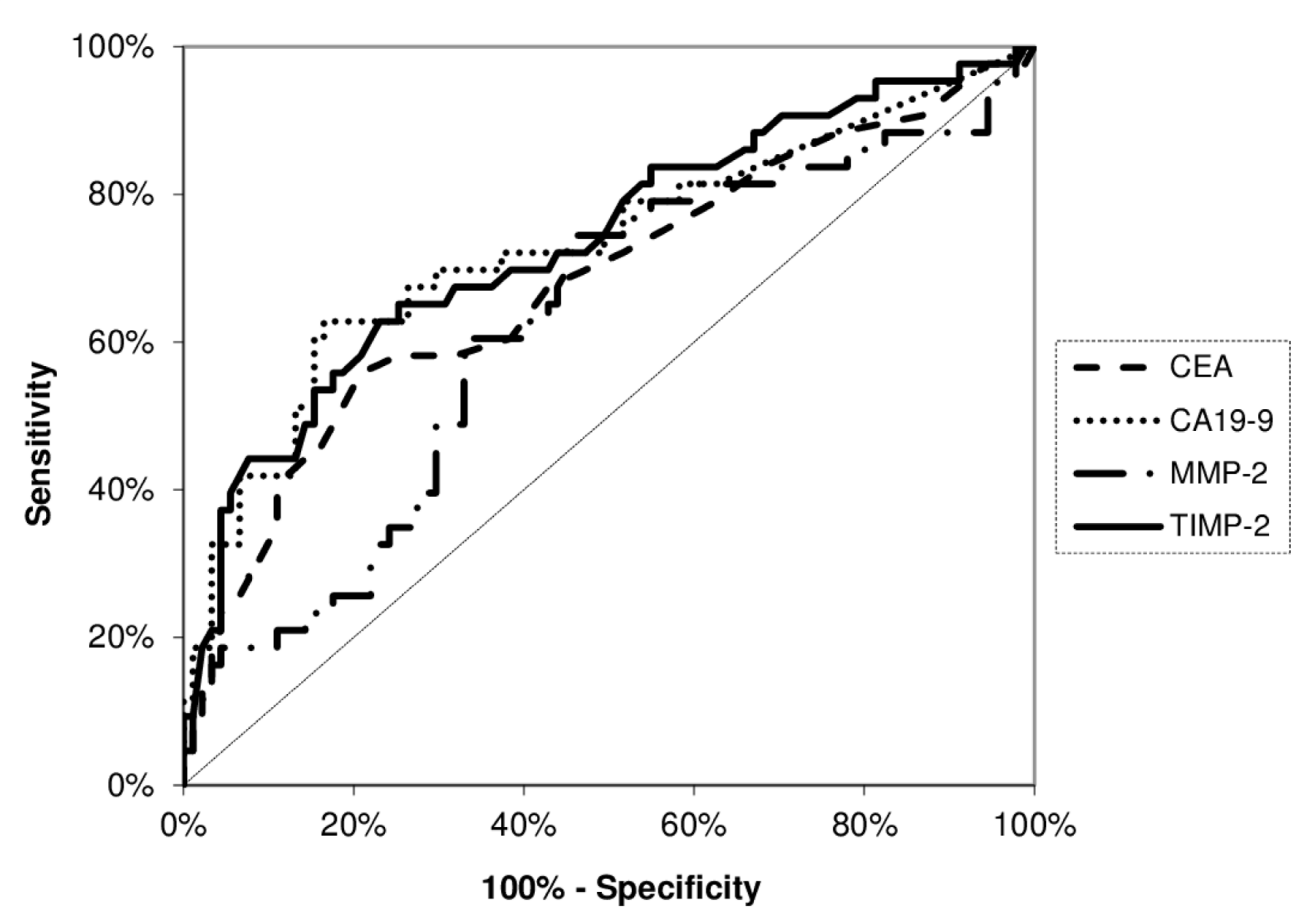
Areas under ROC curve (AUC) for TIMP-2 (0.7340, *p* < 0.001), MMP-2 (0.6218, *p* = 0.023), CA 19-9 (0.7299, *p* < 0.001) and CEA (0.6802, *p* < 0.001) in the differentiation between chronic pancreatitis patients and healthy subjects

The diagnostic significance of MMP-2 and its tissue inhibitor (TIMP-2) was assessed using the diagnostic characteristics, including diagnostic sensitivity and specificity, accuracy, predictive values for negative (NPV) and positive (PPV) results as well as the areas under the ROC curve (AUC). The diagnostic sensitivity of TIMP-2 (79%) was higher than MMP-2 (47%) and classical tumor markers (CA 19-9 – 71% and CEA – 37%). The combined analysis of MMP-2 and TIMP-2 with classical tumor markers (CA 19-9 and CEA) increased diagnostic sensitivity, however the highest value of this parameter was found for combined measurement of MMP-2 and TIMP-2 (96%) (Figure 1). The diagnostic specificity of MMP-2 (67%) was higher than for its tissue inhibitor (37%), but lower that for classical tumor markers – CA 19-9 (97%) and CEA (98%). Similar results were reveled for predictive values for positive (PPV) results and accuracy. Predictive values for negative (NPV) results of TIMP-2 (64%) was higher than for MMP-2 (55%) and CEA (61%), but lower that NPV for CA 19-9 (77%). The area under the ROC curve (AUC) presents the diagnostic usefulness of a biomarker. The AUC for TIMP-2 (0.6037, *p* = 0.013) was higher than for MMP-2 (0.5624, *p* = 0.146), but lower than AUC for classical tumor marker (CA 19-9 – 0.8583, *p* < 0.001; CEA – 0.8790, *p* < 0.001) in the differentiation between PC patients versus healthy subjects (Figure 2). Moreover, in the differentiation between PC and CP patients, the AUC for TIMP-2 (0.6532, *p* = 0.004) was higher than for MMP-2 (0.5378, *p* = 0.47), but lower than AUC for classical tumor marker (CA 19-9 – 0.7765, *p* < 0.001; CEA – 0.7193, *p* < 0.001) (Figure 3). However, in the differentiation between CP patients and the healthy individuals, the AUC for TIMP-2 (0.7340, *p* < 0.001) was the highest among AUC for all proteins tested (MMP-2 – 0.6218, *p* = 0.023; CEA – 0.6802, *p* < 0.001; CA 19-9 – 0.7299, *p* < 0.001) (Figure 4).

## DISCUSSION

Pancreatic cancer is usually diagnosed in late stage of disease, thus more than 90 percent of PC patients die because of this neoplasm [21]. The great challenge of medicine is to find the novel biomarker that will be useful in the early diagnosis of PC patients. The significance of selected MMPs and their tissue inhibitors has already been confirmed in the progression of various gastrointestinal malignancies, such as GC, CC, EC [14–16]. However, little is known about usefulness of these enzymes as potential serum biomarkers in the diagnosis and prognosis of PC patients.

In present study we indicated that the concentrations of MMP-2 in PC patients were higher than in healthy controls and lower in comparison to CP subjects. The serum levels of TIMP-2 in PC patients were significantly lower compared to healthy controls, but statistically higher than in patients with CP. Similar finding was indicated by Singh *et al.*, who determinated that MMP-2 levels were also elevated in PC patients as compared to healthy individuals, however this study was performed on the plasma of PC patients [10]. The immunohistochemical study of Zhai *et al.* has revealed that the expression of MMP-2 was significantly increased in PC samples in comparison to non-tumorous tissues [11]. In addition, Lekstan *et al.* indicated that MMP-2 and TIMP-2 concentrations in PC patients were higher than in CP individuals [13]. Our previous investigations performed on GC, CC and EC patients have revealed that the serum concentrations of MMP-2 and TIMP-2 in cancer patients were statistically lower in comparison to healthy subjects and all these differences reached statistically significance [14–16]. Conflicting findings concerning MMP-2 and TIMP-2 levels in cancer patients presented by different authors might be interpreted by the basic function of MMP-2 and TIMP-2 as well as by the dual role of TIMPs in tumor development. Some investigators suggest, that high levels of TIMP-2 are associated with MMP-2 inhibition, while low concentration of this inhibitor with activation of MMP-2 [22–23]. However, all these investigations confirm that the imbalance between MMP-2 and its tissue inhibitor play an important role in the PC development.

Analysis of MMP-2 and TIMP-2 concentrations according to TNM stage indicated no statistically significant differences between levels of these proteins and tumor stage. However, the serum MMP-2 levels were the lowest in the PC patients with stage II, whereas for TIMP-2 the lowest concentrations were indicated in patients with stage IVb. The statistically significance difference was found only between CEA concentrations and TNM stage. Similar results were found in our previous studies concerning other gastrointestinal neoplasms, where the serum MMP-2 and TIMP-2 levels were not correlated with tumor stage [14–16]. These observations are in the line with the findings of other authors, who also revealed no correlations between plasma concentrations of MMP-2 and TNM stage of PC [10].

In our present study there were no significant associations between concentrations of MMP-2 and TIMP-2 and clinico-pathological characteristic of PC, similar as in results of Singh *et al.* [10]. On contrary, in the investigation of Lekstan, the levels TIMP-2 were significantly higher in patients with nodal involvement (N1) as compared to patients from N0 subgroup [13]. The authors conclude that this molecule may influence on cancer progression and increased invasiveness potential of tumor [13]. On the other hand, the study of Zhai *et al.* has indicated the significant correlation between MMP-2 expression in PC tissue and the presence of lymph node and distant metastases, what was confirmed using immunohistochemical study [11]. Our previous studies performed on GC, CC and EC patients have also revealed that the serum levels of MMP-2 and TIMP-2 did not significantly correlated with clinico-pathological parameters of tumor [14–16].

According to our knowledge, this is the first study comparing diagnostic usefulness of MMP-2 in relation to its tissue inhibitor and classical tumor markers in PC. Based on diagnostic sensitivity we indicated the advantage of TIMP-2 measurement over assessment of MMP-2 and classical tumor markers (CEA and CA 19-9) in the diagnosis of PC patients. Moreover, the combined analysis of TIMP-2 with MMP-2 increased the diagnostic sensitivity up to 96%, and this value was higher than for the measurement of classical tumor markers in combination. In addition, we found that in differentiation between PC versus healthy subjects and PC versus CP, the AUCs for TIMP-2 were larger than the AUCs of MMP-2, while in differentiation between CP and healthy individuals, the AUC for serum TIMP-2 was the highest among all proteins tested. In our previous study, the AUC for other tissue inhibitor – TIMP-1 was also larger than for MMP-9, what may suggest that TIMP-1 has higher diagnostic usefulness than MMP-9 in PC [17].

If we take into consideration the correlations between levels of analyzed biomarker and prognosis of PC patients’ survival, we observed that none of proteins tested was proved to be an independent factor affecting PC patients’ survival. Similar findings were found in our previous investigations, where the serum concentrations of MMP-2 and its tissue inhibitor (TIMP-2) were also not significant prognostic indicators, however these studies were performed on CC [15] and EC [14] patients. Opposite results were indicated in our other study, where we demonstrated that elevated preoperative concentration of other gelatinase – MMP-9 was a significant independent prognostic factor for the PC patients’ survival [17].

## MATERIALS AND METHODS

The study group comprised on 226 individuals, including 92 patients with PC (34 women and 58 men, aged 42–88 years), 43 patients with CP (17 women and 26 men, aged 31–76 years) and control group – 91 healthy voluntaries (60 women and 31 men, aged 21–65 years). The PC and CP patients were diagnosed in the Second Department of General Surgery, Medical University of Białystok, Poland. The clinical diagnosis of PC was based on a microscopic examination of tissue samples and staged using the TNM (tumor-nodulus-metastases) classification, presented by the International Union Against Cancer (UICC) [18]. In addition, cancer patients were divided into following subgroups, based on stage of tumor (TNM), depth of tumor invasion (T factor), the presence of lymph nodes (N factor) and distant metastases (M factor) as well as resectability of tumor and survival (Table 3). Informed consent has been obtained from all the patients and the present project was approved by the Local Ethics Committee (R-I-002/443/2010) of Medical University of Białystok.

**Table 3:** Characteristics of pancreatic cancer (PC) patients

		Number of patients
Group tested	Pancreatic cancer	92
	Chronic pancreatitis	43
	Healthy controls	91
Gender	Female	34
	Male	58
Age	Under 65	42
	65 or above	50
Tumor stage (TNM classification)	II	13
	III	19
	IV	60
Tumor stage (TNM full classification)	II	13
	III	19
	IVA	23
	IVB	37
Tumor size (T factor)	T2	17
	T3	22
	T4	53
Nodal involvement (N factor)	N0	18
	N1	74
Distant metastases (M factor)	M0	55
	M1	37
Resectability of tumor	Resectable	29
	Nonresectable	63
Survival of patients	Alive	13
	Dead	41
	Data not available	38

Serum samples from PC patients were collected prior to the commencement of treatment and stored at −80° C until assayed. Enzyme-linked immunosorbent assay kits (ELISA) were employed to assess the serum concentrations of MMP-2 and TIMP-2 using R&D Systems kits (Abingdon, England) according to the manufacturer’s instructions. The samples were diluted 10-fold before determination of MMP-2 and 50-fold before TIMP-2 measurement. The intra-assay coefficient of variation (CV%) of MMP-2 is reported by the manufacturer as 5.8% at a mean concentration of 18.9 ng/mL, SD = 1.1 and of TIMP-2 as 4.4% at a mean concentration of 1.23 ng/mL, SD = 0.054. Serum concentrations of CA 19-9 and CEA were measured using microparticle enzyme immunoassay kits (MEIA) (Abbott, Chicago, Illinois). The intra-assay CV% for CEA is reported by the manufacturer of the assay kits to be 4.9% at a mean concentration of 2.2 ng/mL, SD = 0.11 and the intra-assay CV% for CA 19-9 – 4.7% at a mean concentration of 38.2 U/mL, SD = 1.80.

The cut-off values for serum levels of MMP-2 (236 ng/mL) and TIMP-2 (100 ng/mL) correspond to the highest accuracy (minimal false-negative and false-positive results) and were determined using Microsoft Office Excel software. The positive results of TIMP-2 are below cut-off value. The reference cut-off values for established tumor markers for PC were calculated based on the 95th percentile (4.0 ng/mL for CEA and 30.0 U/mL for CA 19-9) and established previously in our department by examining blood sera of healthy volunteers [17, 19].

### Statistical analysis

Serum concentrations of MMP-2, TIMP-2, CA19-9 and CEA did not follow a normal distribution in the preliminary statistical analysis (χ^2^-test) and the nonparametric statistical analyses were used. For the comparison between two groups, the Mann-Whitney test was employed. Kruskal-Wallis test was used in the analysis of three or more groups. The post hoc Dwass-Steele-Critchlow-Fligner test was employed, if significant differences were calculated [20]. Moreover, the diagnostic parameters, including the diagnostic sensitivity and specificity, accuracy, predictive value for negative (NPV) and positive (PPV) results for proteins tested were also evaluated. The differences were considered as statically significant when *p* < 0.05. Statistical analyses were carried out using the STATISTICA 9.0 PL program (StatSoft Inc., Tulsa, OK), while diagnostic characteristics and the ROC curves were calculated using Med-Calc statistical software (MedCalc Software, Mariakerke, Belgium) and Microsoft Office Excel program. Univariate analyses of survival were performed using the log-rank test. Multivariate analyses employed Cox’s proportional hazards model.

## CONCLUSIONS

Searching specific tumor biomarkers useful in the diagnosis and prognosis of PC patients is an extremely important task for future medicine. The incidence rate of PC is similar to mortality rate, because this malignancy is usually diagnose in late stage of disease. Thus, novel biomarker that will be assessed using easy to perform, cheap and non-invasive methods is sorely needed. The role of MMPs and their tissue inhibitors (TIMPs) was confirmed in the pathogenesis of many malignancies, including PC. Currently, there are only few data concerning the serum levels of MMP-2 and TIMP-2 in PC patients. However, little is known about the significance of these proteins as potential tumor biomarkers useful in the diagnosis and prognosis of PC in comparison to well-established, classical tumor markers for PC. In our current study we indicated the significant differences between serum levels of TIMP-2 in PC patients, CP individuals and healthy subjects. Opposite relation between MMP-2 and its inhibitor levels may prove the role of the imbalance between these proteins in pathogenesis of PC. In addition, the diagnostic sensitivity of TIMP-2 was the highest among all proteins tested. Moreover, the AUC for TIMP-2 was higher than for MMP-2 in the differentiation between PC patients versus healthy subjects as well as between PC and CP. Presented findings suggest higher clinical usefulness of TIMP-2 than MMP-2 as potential biomarker in the diagnosis of PC patients. However due to discrepancies between associations between MMP-2 and TIMP-2 concentrations and clinico-pathological characteristics of PC as well as survival of PC patients, more studies on large population are needed to support our results.
